# Application of laser speckle flowgraphy to evaluate cerebral perfusion after carotid endarterectomy

**DOI:** 10.1016/j.heliyon.2023.e14400

**Published:** 2023-03-08

**Authors:** Ayako Oi, Hironobu Hayashi, Yasushi Motoyama, Hideaki Kawanishi, Ichiro Nakagawa, Hiroyuki Nakase, Masahiko Kawaguchi

**Affiliations:** a Department of Anesthesiology, Nara Medical University, Japan; b Department of Neurosurgery, Nara Medical University, Japan

**Keywords:** Laser speckle flowgraphy, Ocular blood flow, Carotid endarterectomy, Cerebral hyperperfusion

## Abstract

Cerebral hyperperfusion syndrome (CHS) after carotid endarterectomy (CEA) is devastating, and postoperative monitoring of cerebral perfusion is essential to prevent CHS. We report two cases of successful measurement of ocular blood flow using laser speckle flowgraphy (LSFG) for bedside assessment of the changes in cerebral perfusion after CEA. An 18.7% (case 1) and 47.7% (case 2) increase in ocular blood flow were measured postoperatively using LSFG compared with the baseline. LSFG might be applicable to evaluate cerebral perfusion after CEA.

## 1 Introduction

Carotid endarterectomy (CEA) is the standard surgery for stroke prevention in patients with severe internal carotid artery (ICA) stenosis [1,2]. Reperfusion of the cerebral tissue after the release of the carotid artery clamping can lead to cerebral swelling and/or hemorrhage, the neurological symptoms of which are known as cerebral hyperperfusion syndrome (CHS) [3]. CHS is a rare but disastrous complication of CEA [4]. The incidence of CHS after CEA has been reported to be 1%–3.2% [5,6]. Cerebral monitors, including transcranial Doppler sonography (TCD) and regional cerebral saturation of oxygen (rSO_2_) measured using near-infrared spectroscopy (NIRS), are used clinically in the perioperative management of CEA to predict CHS [7].

There have been several reports on the intraoperative use of laser speckle flowgraphy (LSFG) to assess the ocular blood flow during cardiovascular surgery and CEA as a cerebral perfusion monitor [[8], [9], [10], [11]]. LSFG is a less invasive technique that can detect ocular blood flow contributed by the ophthalmic artery, which is the first main branch of the ICA [12]. Motoyama et al. [12] revealed that the reduction ratio of ocular blood flow with LSFG due to carotid clamping was significantly correlated with the change in rSO_2_. To our knowledge, this is the first report on ocular blood flow monitoring using LSFG after the release of carotid clamping in CEA.

## 2 Case report

### 2.1 Methods

This case report was approved by the institutional review board of Nara Medical University (approval number: 1219). Written informed consent was obtained from both patients who participated in this case report.

#### 2.1.1 Principles of LSFG

LSFG (Softcare Co., Ltd., Fukuoka, Japan) evaluates the perfusion in the optic nerve head and choroid using the laser speckle phenomenon. LSFG has the advantage of non-contact quantitative estimation and the ability to repeatedly measure the ocular perfusion during surgery.

The LSFG system consists of a diode laser-equipped fundus camera and a conventional charge-coupled device camera. The LSFG device was used to evaluate the intraoperative ocular blood flow with a focus on the optic nerve head on the affected side in these cases. The basic principles of LSFG have been described in previous reports [13,14]. LSFG can quantitatively estimate the perfusion of retina in a non-invasive manner by utilizing the laser speckle phenomenon, which is an interference phenomenon that occurs when a coherent light source scatters on a diffuse surface. Depending on the movement of the blood cells in the tissue (i.e., blood flow), the structure of the speckle pattern that appears under laser irradiation changes rapidly; thus, the pattern varies depending on the blood flow rate. The mean blur rate (MBR) calculated from this blurring variation is a quantitative indicator of the relative blood flow velocity.

#### 2.1.2 Measurement of the ocular blood flow for the assessment of cerebral perfusion

**Fig. 1:**
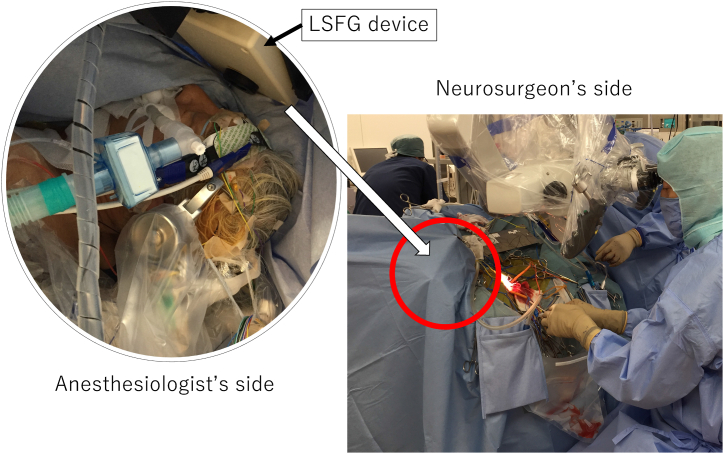
Intraoperative use of LSFG This device consists of a fundus camera equipped with a diode laser and an ordinary charge-coupled device camera. LSFG enables noncontact quantitative estimation and repeated measurement of ocular perfusion during surgery. LSFG: laser speckle flowgraphy.

Impaired ocular perfusion, i.e., reduced cerebral blood flow, occurs because the ophthalmic artery arises from the carotid artery. The ocular blood flow measured by LSFG has been used for monitoring cerebral blood flow [[10], [11], [12],^15^]. The pupil of the eye on the affected side was dilated with 0.5% tropicamide and 0.5% phenylephrine hydrochloride (Mydrin-P ophthalmic solution; Santen Pharmaceutical Co., Ltd., Osaka, Japan) before LSFG measurement. The eye was opened when measuring the MBR of the optic nerve head using LSFG (Fig. 1). Continuous measurement of the optic nerve head MBR using LSFG was not performed in these cases to avoid corneal damage due to corneal dryness.

#### 2.1.3 Intraoperative assessment of cerebral perfusion

MBR of the ocular blood flow was measured using LSFG to assess the cerebral perfusion during CEA, and its increase and reduction ratios between the baseline and the other time points were calculated. During CEA, MBR was measured seven different times using LSFG. The ocular blood flow was measured using LSFG at the following points: 1) as a control before starting the surgery with the patient placed on the operating table and the head fixed by the skull clamp (T1); 2) after the exposure of the carotid artery before carotid clamping as a baseline (T2); 3) after carotid clamping to calculate the reduction ratio (T3); 4) when the shunt tube was placed in the carotid artery (T4); 5) after removing the shunt tube before the complete repair of the carotid artery (T5); 6) after the release of carotid clamping, followed by complete plaque removal and angiography to confirm that blood flow of the carotid artery had restarted (T6); and 7) at the end of surgery (T7). In case 1, T4 and T5 were omitted as a shunt tube was not used.

#### 2.1.4 Postoperative assessment of cerebral perfusion

The LSFG device was used to evaluate MBR on the affected side at the bedside 1 or 2 days after CEA. Ocular blood flow measurement using LSFG was performed after dilating the pupil of the eye on the affected side without sedation.

In the assessment of CBF after CEA, an ipsilateral increase of >100% detected using single-photon emission computed tomography (SPECT), compared with that at baseline, is related to cerebral hyperperfusion [16,17].

#### 2.1.5 Case 1

A 65-year-old man was coincidentally diagnosed with ICA stenosis during magnetic resonance imaging (MRI) examination following radiation therapy for laryngeal cancer. Magnetic resonance angiography (MRA) and carotid angiography revealed asymptomatic 90% stenosis in his left ICA according to the North American Symptomatic Carotid Endarterectomy Trial criteria (NASCET). N-isopropyl-p-[123I]-iodoamphetamine SPECT (IMP-SPECT) showed decreased cerebral blood flow in the left middle cerebral artery territory. Ophthalmologic examination indicated that the visual acuity and visual field were normal.

MBR measured by LSFG at T1, T2 (baseline), T3, T6, and T7 was 13.0, 18.7, 16.8, 16.9, and 18.7, respectively. The patient was extubated after the surgery, transported to the ICU, and managed under light sedation with a 0.4 μg/kg/h dose of dexmedetomidine. SBP was maintained under 140 mmHg with 3–5 mg/h of nicardipine. The ocular blood flow was measured using LSFG and SPECT on the first postoperative day. MBR measured using LSFG was 22.2, and its increase ratio was 18.7%. IMP-SPECT revealed a 5–10% increase in cerebral blood flow in the left middle cerebral artery (MCA) territory. Significant cerebral hyperperfusion was not observed. Although his hospital stay period was extended because of the reoperation due to postoperative bleeding from the wound on the third postoperative day, he was discharged on the 15th postoperative day without any neurologic deficits.

#### 2.1.6 Case 2

**Fig. 2:**
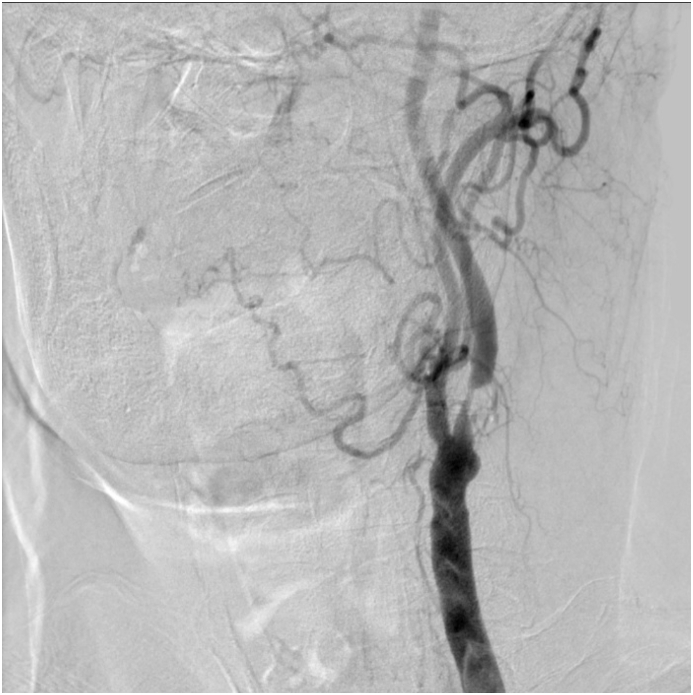
MRA of case 2 MRA and carotid angiography shows 75.1% ICA stenosis as per the NASCET. MRA: magnetic resonance angiography, ICA: internal carotid artery, NASCET: North American Symptomatic Carotid Endarterectomy Trial criteria.

**Fig. 3:**
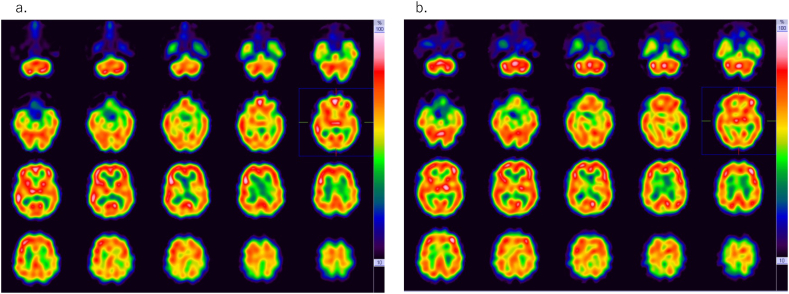
IMP-SPECT before (a) and after (b) CEA in case 2 Compared with preoperative SPECT, postoperative SPECT (b) shows a 5%–10% increase in the cerebral blood flow in the left MCA territory (a). SPECT: single-photon emission computed tomography, IMP-SPECT: N-isopropyl-p-[123I]-iodoamphetamine SPECT, CEA: carotid endarterectomy, MCA: middle cerebral artery.

Severe left ICA stenosis was observed in the carotid artery in a 74-year-old man with a history of chronic kidney disease, hypertension, and dyslipidemia during his routine examination. He did not have any symptoms of ischemic syndrome; however, asymptomatic cerebral infarction was also detected via MRI. MRA and carotid angiography revealed 75.1% ICA stenosis as per the NASCET (Fig. 2). IMP-SPECT revealed decreased cerebral blood flow in the left middle cerebral artery territory (Fig. 3a). Ophthalmologic examination indicated that the visual acuity and visual field were normal.

**Fig. 4:**
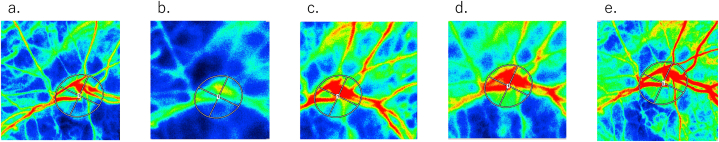
The changes in MBR at T2 (a), T3 (b), T4 (c), T6 (d), and T7 (e) in case 2 MBR shows decreased ocular blood flow due to carotid artery clamping (b: T3) and increased ocular blood flow due to reperfusion (c: T4, d: T6 and e: T7) compared with baseline (a: T2). MBR: mean blur rate.

**Fig. 5:**
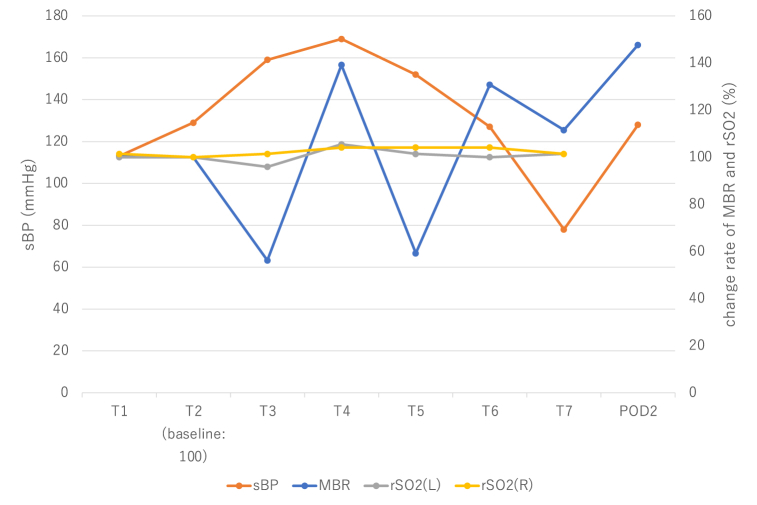
Changes in MBR, systolic arterial blood pressure, and rSO_2_ measured using NIRS. The MBR and rSO_2_ measured using NIRS of T2 (baseline) was set as 100 and the percentage increase was expressed for each time. MBR: mean blur rate, rSO_2_: regional cerebral saturation of oxygen, NIRS: near-infrared spectroscopy.

MBR measured using LSFG at T1–T7 were 13.1, 13.0, 7.3, 18.1, 7.7, 18.4, and 14.5, respectively. Fig. 4, Fig. 5 show the changes in MBR, SBP, and rSO_2_ measured using NIRS. After the surgery, the patient was extubated and transferred to the ICU. On the second postoperative day, MBR measured using LSFG was 19.2, and its increase ratio was 47.7%. On the fourth postoperative day, IMP-SPECT revealed a 5–10% increase in cerebral blood flow in the left MCA territory. Significant cerebral hyperperfusion was not detected (Fig. 3b). He was discharged on the seventh postoperative day without any neurologic deficits.

## 3 Discussion

We reported two cases of successful measurement of ocular blood flow using LSFG for postoperative bedside assessment of the changes in cerebral perfusion after CEA. Increases of 18.7% (case 1) and 47.7% (case 2) in MBR, compared with the baseline MBR recorded before intraoperative ICA clamping, were measured using LSFG. CBF improved in both cases after CEA, and CHS was not evident in the SPECT or clinical presentations.

CHS is a well-known postoperative complication of CEA with high morbidity and mortality. This syndrome is characterized by a constellation of symptoms and findings that may include ipsilateral headache, seizures, hypertension, focal neurologic deficits, and intracerebral hemorrhage. The syndrome occurs rarely but has a potentially fatal mortality rate of 38.2–50.0% [5].

LSFG is a less invasive technique that can detect ocular blood flow. LSFG is unaffected by muscle blockade and inhaled anesthetics [10,11] and can convert the ocular flow velocity into continuous fluctuations in real-time [[9], [10], [11],^18^]. Moreover, LSFG monitors the blood flow volume as well as velocity [19]. Hecht N et al. [18] revealed that LSFG can be used to assess CBF on the brain surface before and after superficial temporal artery to middle cerebral artery bypass grafting. Hayashi H et al. [10] evaluated the validity of the ocular blood flow measured using LSFG for the assessment of cerebral perfusion during aortic arch surgery. LSFG can be used for intraoperative CBF monitoring. Moreover, since the LSFG device is portable, it can also be used for bedside CBF monitoring after CEA, similar to point-of-care testing. In our cases, the LSFG device did not interfere with the surgical procedure during intraoperative use and was easily used at the bedside after surgery.

Case 2 presumably has a lower cerebral circulatory reserve compared to case 1 because ICA clamping caused a larger decrease ratio of 43.8% in ipsilateral ocular blood flow values (MBR). Previous report [20] showed that insufficient collateral flow indicated a low cerebral circulatory reserve, which is one of the risk factors for postoperative CHS. On the second postoperative day, relatively greater increase ratio of 47.7% in MBR was observed in Case 2. In the present case series, it was observed that the degree of increase in postoperative MBR tended to be greater in patients with low cerebral circulatory reserve with the use of LSFG. LSFG, which allows intraoperative assessment of cerebral circulatory reverse as a numerical value, may be useful for risk classification of patients with excessive increase in postoperative cerebral perfusion.

Several cerebral monitoring techniques have been used for early detection of CHS during surgery and postoperative management of cerebral blood flow in ICU [7]. In particular, TCD and rSO_2_ measured using NIRS are useful for assessment cerebral perfusion. However, these monitoring techniques are associated with some limitations in terms of use, principles or interpretation. Before and after operation, SPECT can directly evaluate the changes in CBF after CEA and assess the risk of CHS after CEA [4,21]. However, SPECT cannot be used for bedside monitoring and involves substantial costs and technical complexity, which limit its clinical availability [22].

Cerebral hyperperfusion is defined as a postoperative increase of more than twice the preoperative value of CBF detected by SPECT [23]. The TCD diagnostic criteria proposed greater than twice the preoperative mean blood flow velocity of the affected middle cerebral artery [23]. However, the LSFG criteria for cerebral hyperperfusion are unknown at this time. Since LSFG is primarily a blood flow velocity monitor, a 100% increase based on the TCD criteria could be applicable to the LSFG criteria. Further studies would be required to define cerebral hyperperfusion based on LSFG.

There are several limitations to this case report. First, there is a difference in the postoperative MBR rate of increase between case 1 and case 2, although the increase in the ratio of CBF detected using SPECT in both cases was almost the same, presumably because the postoperative measurement of MBR using LSFG and SPECT were obtained on different dates in case 2, unlike case 1 where it was obtained on the same day in case 1. In addition, there were no significant changes in blood pressure or neurological symptoms on both days, comparison of LSFG and SPECT in case 2 is reliable. Second, continuous monitoring of MBR in the optic nerve head using LSFG carries the risk of corneal damage as the eyelid is maintained open. Therefore, the use of LSFG is inevitably limited to intermittent use. Third, it may be difficult to obtain accurate MBR data in the optic nerve head using LSFG in patients with severe cataracts as it may not be possible to observe the fundus perfusion. Fourth, the LSFG criteria for CHS are unknown at this time. Further studies with larger sample sizes and using strict evaluation of the relationship between the ocular blood flow velocity and CBF measured using LSFG and SPECT after CEA are required in the future.

In summary, we report two cases of successful measurement of ocular blood flow using LSFG for bedside assessment of the changes in cerebral perfusion after CEA. An 18.7% (case 1) and 47.7% (case 2) increase in ocular blood flow were measured postoperatively using LSFG compared with the baseline. LSFG might be applicable to evaluate cerebral perfusion after CEA.

## Author contribution statement

All authors listed have significantly contributed to the investigation, development and writing of this article.

## Funding statement

This research did not receive any specific grant from funding agencies in the public, commercial, or not-for-profit sectors.

## Data availability statement

Data included in article/supplementary material/referenced in article.

## Declaration of interest's statement

The authors declare no conflict of interest.
